# Gambling Disorder during Dopamine Replacement Treatment in Parkinson's Disease: A Comprehensive Review

**DOI:** 10.1155/2014/728038

**Published:** 2014-07-08

**Authors:** Domenico Pirritano, Massimiliano Plastino, Domenico Bosco, Luca Gallelli, Antonio Siniscalchi, Giovambattista De Sarro

**Affiliations:** ^1^Department of Neuroscience, “S. Giovanni di Dio” Hospital, 88900 Crotone, Italy; ^2^Department of Health Science, University of Catanzaro, 88100 Catanzaro, Italy; ^3^Department of Neuroscience, “L'Annunziata” Hospital, 87100 Cosenza, Italy

## Abstract

Gambling Disorder (GD) is characterized by “the failure to resist gambling impulses despite severe personal, family or occupational consequences”. In the fifth edition of the Diagnostic and Statistical Manual of Mental Disorders (DSM-V), GD replaces the DSM-IV diagnosis of Pathological Gambling (PG). GD estimated prevalence ranges between 0.4% and 3.4% within the adult population and it seems to be more common in patients with Parkinson's disease (PD). In this population, GD recently has become more widely recognized as a possible complication of dopamine agonist (DA) therapy. This association has aroused great interest for the dramatic impact GD has on patients' quality of life. Management of PG in patients with PD could be demanding. It is based on patient and caregiver education, modification of dopamine replacement therapy, and in some cases psychoactive drug administration. In this review article, the authors provide an overview of GD pathogenesis during DA therapy as well as a summary of available treatment options.

## 1. Introduction

Gambling Disorder (GD) is characterized by “the failure to resist gambling impulses despite severe personal, family or occupational consequences.” In the fifth edition of the Diagnostic and Statistical Manual of Mental Disorders (DSM-V), GD replaces the DSM-IV diagnosis of Pathological Gambling (PG) [1]. DSM-IV classified this disorder as an Impulse Control Disorder (ICD) [2]. GD differs from PG in that it requires 4 rather than 5 criteria for diagnosis and excludes the “Illegal Acts” criterion [1]. The DSM-5 work group moved PG to the category “Addiction and Related Disorders” [3]. The rationale for this change is that the growing scientific literature on PG reveals common elements with substance use disorders. Brain imaging studies and neurochemical tests have made a “strong case that [gambling] activates the reward system in much the same way that a drug does” [4].

GD estimated prevalence ranges between 0.4% and 3.4% within the adult population [5–7]. GD, along with compulsive sexual behaviour, compulsive buying, the addiction-like compulsive use of dopamine replacement therapy, or dopamine dysregulation syndrome (DDS) [8], seems to be more common in patients with Parkinson's disease (PD) than in the general population [9]. GD is reported as a side effect of dopamine agonist (DA) therapy used in PD [10, 11], with a dramatic impact on the quality of life of patients and their caregivers. This review describes some aspects of GD pathogenesis during DA therapy and its management.

## 2. Epidemiology and Risk Factors

GD prevalence in North America is reported to be between 0.4% and 1.9% within the adult population [10, 12–14]. In PD, some evidence suggests that GD is associated with an early onset disease, longer disease duration and high novelty seeking personality traits [10, 15, 16]. Other independent risk factors include younger age, male sex, cigarette smoking, prior personal or family history of alcohol addiction and impulse traits [17–20]. According to the available data, GD prevalence rates in PD may vary considerably, ranging from 6% in PD patients not receiving DA to 17% among those on DA treatment [21]. In PD patients under DA therapy, concurrent levodopa use increases the risk to develop GD by approximately 50% [17]. GD involves a subset of patients only, suggesting an underlying susceptibility, mediated by PD-specific factors such as a dysregulation of dopaminergic system, which may also modulate underlying temperament traits. The psychological profile of PD patients may have a role as a risk factor, since impulse sensation seeking personality traits and addiction proneness characterized PD patients who develop GD.

Some authors suggest that DA, but not L-dopa treatment, may worsen executive functions in patients affected by early/mild PD [22]. DAs, compared with L-dopa, have significantly greater affinity for D3 receptors (approximately 20 to 100 times more affinity for D3 than D2), and little or no affinity for D1 receptors [23].

Voon et al. observed that GD was associated with DAs but not with agonist subtype or doses: both D1/D2 (pergolide) and D2/D3 (ropinirole and pramipexole) agonists were equally implicated [10, 21]. However, the authors do not rule out D3 mechanisms, given that pergolide may have greater D3 than D1 receptor affinity [24]. Other authors confirmed these data, finding that agonist dose and duration were non-significant.

No differences were observed between pramipexole, ropinirole, and pergolide in their association with GD [25], and DA doses did not predict GD development [26].

Thondam and coworkers reported a case of a young patient that developed severe, socially disruptive impulsivity manifesting with pathological gambling during a long-term bromocriptine therapy [27].

Other retrospective reports suggest a different role of specific dopamine receptor agonists, considering their different dopamine receptor affinity [28, 29]. These authors found an increased prevalence of GD in PD patients treated with pramipexole, compared with other dopamine receptor agonists. In these patients, GD may develop for an excessive stimulation of D3 receptors.

The role of DA dose in increasing GD risk is still not clear [19, 30]. Perez-Lloret et al., documented that PD patients with impulse-control disorder symptoms were exposed to higher dopamine doses than those without them (1.6 ± 0.1 versus 1.0 ± 0.1 daily-defined doses). However, using a dose-response pharmacodynamic model authors disclosed a significant nonlinear dose-response relationship between dopamine agonists and frequency of ICD symptoms [31].

Moreover, recently in a retrospective study performed on 20 patients with PD, Castrioto and coworkers documented that high chronic dopaminergic treatment (mean levodopa equivalent daily doses 1420/mg) induced pathological hyperdopaminergic behaviours in 8/20 patients, which had resolved in 7/8 patients when the dosage was reduced (mean levodopa equivalent daily doses 320/mg) [32].

Mood disorders also represent powerful risk factors, and low performance in cognitive tasks requiring frontal function has been associated with GD [15, 33, 34].

## 3. Clinical Manifestations

GD is defined as failure to resist gambling impulses despite severe consequences on personal, family or professional life. It is often under-recognized: patients very rarely give spontaneous information about it, do not understanding that GD may be related to PD treatment [35].

Lives of patients affected by GD become dominated by gambling behaviour, leading to overwhelming financial burdens, inability to maintain a career, and eventually the disintegration of family relationships [36]. The most frequent attitudes are slot machines, lottery scratch cards and bingo, and GD occurs more frequently during the “on” phase [37].

## 4. GD: Neurotransmitters and Pathophysiology

GD shares diagnostic features with substance use disorder (SUD), and its pathophysiology involves specific neurotransmitter systems, brain regions and neural circuitries. The main neural pathways seem to be corticostriato-thalamo-cortical circuitry and mesocorticolimbic pathway for reward and reinforcement processes.

### 4.1. Neurotransmitters

Dopamine (DA), a neurotransmitter implicated in reward and reinforcement, is probably involved in GD pathogenesis and some authors used positron emission tomography (PET) to test whether GD is associated with abnormalities in D2 and D3 receptor levels, as observed in SUD. They compared D2 and D3 receptor binding between subjects with and without GD, and assessed the relationship between binding profiles, impulsiveness (a known predisposing trait) and gambling severity. Unlike with substance use disorder, there appear to be no marked differences in D2/D3 levels between healthy subjects and pathological gamblers, suggesting that low receptor availability may not be a necessary feature of addiction. However, relationships between [11C]-(+)-PHNO binding and gambling severity/impulsiveness suggests involvement of the D3 receptor in impulsive/compulsive behaviours. These authors suggest that strategies focused on the D3 system may be effective in treating some individuals with GD [38].

Several functional imaging studies provided further evidences about the involvement of specific brain regions in PG behaviours. Areas such as prefrontal cortex (ventromedial and orbitofrontal areas), ventral striatum (nucleus accumbens) and amygdala showed a reduced activation in pathological gamblers during fMRI studies, suggesting a relationship with aberrant reward and response inhibition [39]. In another fMRI study about motivational and emotional states in men with and without PG, subjects with PG reported stronger gambling urges and showed relatively reduced activation of frontal cortical, basal ganglionic and thalamic brain regions while viewing gambling tapes, during the period prior to the onset of subjective motivational or emotional response [40]. In a [11C] raclopride positron emission tomography (PET) study, Steeves et al. assessed dopaminergic functions during gambling in PD patients. PG patients demonstrated a greater reduction in binding potential in the ventral striatum during gambling compared with control subjects, reflecting greater dopaminergic release. Similar findings are reported in subjects with chemical addictions [41]. In a recent study, the authors found that PD patients with PG have abnormal resting state dysfunction of the mesocortico-limbic network on SPECT imaging, possibly associated with a drug-induced overstimulation of relatively preserved reward-related neuronal systems [42]. All these findings, based on different functional imaging studies, show that PG shares many features with drugs addiction such as the relation with a deficiency of the mesolimbic dopaminergic reward system.

Most recently, neuroimaging has provided new evidence that increased susceptibility for ICD and addiction associated with the impulsive personality trait may be dependent on normal variations in brain function that, in PD, interact with DA agonist exposure to produce pathological behaviours [43].

Similarly to what has been observed in drugs addicts, a reduced activation in prefrontal cortex (ventromedial area) has been observed in patients with GD. These data support the view of GD within the spectrum of behavioural addictive disorder.

The development of GD in patients receiving low doses of dopaminergic drugs suggests that a genetic predisposition may play a role in some cases [44]. Lee et al. described a variant of the serotonin 2A receptor gene (HTR2A) associated with GD in PD patients receiving DA therapy, mainly those taking low doses of dopaminergic drugs [45].

Other neurotransmitters may have a role in GD pathophysiology. Serotonin (5-HT) has been implicated in control over motivated behaviours. Abnormalities in 5-HT function have been described in subjects with PG [46]. Dopamine has been involved in reinforcing and rewarding behaviours, and it has long been associated with these processes in drug addiction [6].

Norepinephrine (NE) system has been involved in drug relapse, reward and sensitization, and high NE levels have been observed in CSF and urine samples of subjects with GD as compared to controls [47, 48]. A decrease of GABAergic neurotransmission system is involved in the expression of behavioural impulsivity [49–51].

### 4.2. Pathophysiology

In scientific literature there are different hypotheses for the association between GD and dopaminergic stimulation in PD patients.

Even if D1, D2 and D3 receptors are involved in motor responses, the activation of D1 and D2 receptors, located in the dorsal striatum, is associated mainly with the motor effects of the medications. In contrast, the activation of D3 receptors located in mesolimbic pathways and in areas such as nucleus accumbens and olfactory tubercle is involved in motivation and reward behaviours [23]. GD may be due to an excessive stimulation of this D3 receptor subtype [29, 52–55].

PD is characterized by a massive loss of dopaminergic neurons in the substantia nigra, with a pronounced depletion of dopamine in the nigrostriatal pathway and a decreased stimulation of the striatal D1 and D2 receptors [56]. This depletion leads to disturbance in the cognitive, limbic, and associative corticobasal gangliathalamo-cortical loops, and might predispose to the occurrence of GD in PD. PD patients, even in the absence of dementia or depression, are likely to show a range of clinically significant impairment in executive functions, most probably linked to degeneration in the basal ganglia-thalamo-cortical circuits (striatal-frontal tracts), secondary to cell loss within the substantia nigra (SN) (due to decreased dopaminergic transmission in fronto-striatal loops) [57, 58].

L-dopa, in this stage of disease, may improve certain cognitive functions that are associated with the severely depleted dorsal striatum, while at the same time impairing other cognitive functions, associated with the relatively intact ventral striatum [59]. Thus, one explanation is that excessive, targeted dopamine stimulation of intact ventral striatal receptors in early or mild PD leads to an “overdose” of ventral striatal-cortical circuitry that can manifest itself in the clinical phenomenon of impulsive-compulsive behaviours, such as GD. These behaviours are maintained by ongoing dopaminergic stimulation of a sensitized ventral striatal system, which is manifested clinically as an increased drive for certain behaviours and maintained by an inability to learn from negative decision outcomes [60].

Therefore, all these data have provided insight into the neurochemical, neuroanatomical and functional basis of GD developing in PD patients showing that DA agonists may induce changes in brain function that impair patients' ability to learn appropriately from both reward and punishment.

## 5. GD Management in Patients with PD

GD management in patients with PD is challenging and there are limited data to support any particular therapeutic strategy. Its association with DA therapy suggests that dopaminergic treatment modifications may be effective. Compulsive behaviours often resolve after DA tapering, switching to a different agonist or discontinuing DA entirely [28, 29, 61].

Some authors reported that 80% of patients discontinuing or significantly decreasing DA doses, or switching to a different agonist, experienced full or partial remission of GD symptoms [29, 62]. Many PD patients are reluctant to discontinue DA treatment because of the motor benefits associated with their use. Moreover, when reducing DA doses, a withdrawal syndrome (DAWS) may occur in a subset of patients, causing profound disability [63].

The first step in GD management is to try to prevent it. Before starting DA therapy, risk factors such as male sex, young age and a history of drug abuse should be taken into consideration. Another aspect is to identify subjects with GD, also involving caregivers.

Very limited data supporting the use of psychiatric drugs for GD in PD exist. The neurobiological similarities between GD and substance use disorders suggest that specific pharmacotherapies may be helpful in treating GD.

Atypical antipsychotics, antidepressants, mood stabilizers and various psychosocial interventions have been proposed to treat GD in patients with PD [28, 64]. The role of these various agents in the management of GD is not well established, and only few case reports have been published on this topic [8].

### 5.1. Serotonin Reuptake Inhibitors (SSRIs)

Serotonin system has long been associated with impulse control and different studies support its role in GD [39, 65]. Decreased serotonin function within ventral medial prefrontal cortex may cause disinhibition and contribute to GD development. Drugs affecting serotonin neurotransmission may represent potential treatment for GD.

SSRI, though effective in obsessive-compulsive disorders, provide questionable benefit in GD, since they may facilitate dopaminergic transmission and could worsen gambling attitude. In one trial on fluvoxamine versus placebo, this drug was associated with a statistically significant improvement in GD [66], but this data was not confirmed in another study [67]. Paroxetine effect on GD was analyzed in two studies. The first one showed a significant effect of paroxetine on GD, evaluated with the Gambling Symptoms Assessment Scale [68]. The other one failed to demonstrate a significant benefit [69]. Sertraline did not prove to be superior to placebo [70]. Citalopram seems to be effective on GD in the general population [71]. SSRIs could have some short-term efficacy on GD in the general population, so they may be helpful in patients with co-occurring anxiety or mood disorders. Existing reports on the efficacy of SSRI for GD treatment in patients with PD have not been encouraging [72].

### 5.2. Mood Stabilizers and Antipsychotics

Lithium and valproate, an antiepileptic drugs that increase the GABA levels [73, 74], have been reported to be effective on gambling and manic symptoms compared to placebo in patients with GD and bipolar disorders [64, 75]. Recently patients with a dopamine dysregulation syndrome, a pathological condition that contribute to behavioral disturbances, and who were all refractory to medication adjustments responded by the addition of valproic acid [76].

There are some case reports on atypical antipsychotics usage in GD treatment. Low-dose risperidone may be effective in controlling GD behaviour in PD patients [11, 29, 54]. A positive effect of high-dose quetiapine in controlling gambling behaviour in a patient with PD have been observed [77]. N-desmethylclozapine, the major active plasma metabolite of the atypical antipsychotic clozapine has a potent partial agonist activity on dopamine D2/D3 receptors [78, 79]. Some authorsreport the effectiveness of clozapine on persistent gambling behaviour following discontinuation of DA therapy [72, 80, 81], although its use requires careful monitoring due to potential risk of agranulocytosis [78].

Controlled studies on atypical antipsychotic drug for GD in non-PD subjects showed that olanzapine is not effective in gambling behaviour treatment [65, 82, 83]. Some authors found that aripiprazole, a partial dopamine D2/D3 receptor agonists, may be effective in treating impulsive/compulsive and addictive behaviours via regulation of reward pathway circuitries [84, 85], although Cohen et al. reported in 3 non-PD patients that aripiprazole may induced PG [86]. Recently, Gaboriau and coworkers in a retrospective study performed in 166 non-PD patients with history of PG reported that in 8 of these patients, aripiprazole induced the development of PG that improved after its dismission [87]. In PD patients, aripiprazole-treatment may be related to exacerbation of motor symptoms [88]. A randomized trial on bupropion, a drug with monoamine reuptake inhibition and nicotinic receptor antagonism properties, failed to demonstrate its efficacy in GD [89].

Topiramate may play a role in GD treatment. It has been known to have a positive effect on binge eating disorder associated with obesity, and compulsive impulsive sexual behaviours in patients with psychiatric disorders [90, 91]. Topiramate has multiple mechanisms of action, in particular it is able to bind the GABAA receptors increasing the GABA levels with a concentration-dependent effect [73, 74, 92] and recently it has been shown to inhibit levodopa-induced dyskinesia in animal models, suggesting a possible inhibitory effect on dopaminergic drugs [93]. In a recent case report, the authors suggest that topiramate may be an effective therapy in PD patients with PG [94].

An open non-randomized trial on zonisamide in fifteen PD patients with GD demonstrated a marked reduction in impulsive behaviour severity, without clinically significant side effects [95].

### 5.3. Opioid Antagonists

Dopaminergic systems that influence rewarding and reinforcing behaviours have been implicated in GD. Gambling triggers dopamine release, which in turn may reinforce the pathological behaviour [96].

Opioid receptor antagonists are thought to decrease dopamine neurotransmission in the nucleus accumbens and in the motivational neurocircuitry. Their efficacy have been studied in PG treatment for their indirect modulation of mesolimbic dopamine circuitry and their role in alcohol and opiate dependence treatment [96].

In non-PD patients, a positive effect of naltrexone in PG treatment was demonstrated in a double-blind, placebo-controlled trial, with a statistically and clinically significant difference. Naltrexone was more effective in gamblers with more severe urges than in those who describe their urges to gamble as moderate [97]. An open-label study suggested naltrexone efficacy in reducing urge intensity to gamble when given in high doses (50 to 250 mg/day) [98]. These data were confirmed in another study in which naltrexone was administered at doses typically used in alcohol or opiate dependence, with a good safety profile [99]. However, clinical use of naltrexone is limited by its side effects. In a case report series, the authorsobserved that naltrexone could be an effective option for GD treatment in PD patients who develop GD after DA therapy [100].

Nalmefene, another opioid antagonist, has been found to be effective in non-PD subjects with GD, but its efficacy is connected with dosage [101]. Another multicentre randomized controlled trial demonstrated that low dose nalmefene (25 mg/day) is effective on GD symptoms in the short-term, with few adverse events and without the dose-dependent hepatotoxicity of naltrexone [102].

### 5.4. Behavioural Therapies

In addition to pharmacological treatments, psychosocial interventions may be considered in GD management. Involving caregivers in the management of GD may be useful. Counselling and limiting access to money and medications in conjunction with tapering DA treatment have been effective in some patients [35].

Several non-pharmacological treatments have been studied in GD, such as behavioural, cognitive, and psychoanalytic therapy. Cognitive behavioural therapy or attendance at Gamblers Anonymous meetings may play a role in selected groups of patients with GD, having been associated with better outcome in non-PD subjects [103]. They were seen effective on gambling severity and frequency, and these effects were maintained over time [104, 105]. However, their effectiveness have not been examined in subjects with PD.

### 5.5. Deep Brain Stimulation

Deep brain stimulation (DBS) surgery of the subthalamic nucleus (STN) or globus pallidus internus may markedly improve “off”-medication motor symptoms, and STN DBS has the potential to allow significant reduction in drug dose [106]. Therefore, STN DBS could be seen as a treatment option for patient with dopaminergic drug related behaviours.

However, the relationship between DBS and GD seems to be complex. Few case reports and small case series have reported contrasting effects of STN DBS on dopamine misuse and GD, while a recent prospective study found clear beneficial effects of STN DBS on these disorders.

The efficacy of STN stimulation for GD in PD patients has not been fully clarified. Existing data are contradictory. Some case series suggest that bilateral DBS of STN may improve GD, allowing a decrease in levodopa dose or DA discontinuation [107]. Other evidence shows that GD may begin in the early postoperative period, and 71% of patients with pre-operative GD remained unimproved or worsened post-operatively [108–111]. Up to date, ICDs should not be considered an indication for DBS.

In an observational study on 110 consecutive parkinsonian patients scheduled for STN DBS surgery, Eusebio et al. suggest that STN DBS may reduce compulsive use of dopaminergic medication and its behavioural consequences. Whether this improvement is the result of STN DBS or the consequence of better treatment management remains to be established. Most of the addictive behaviours improve after STN DBS partly as a result of the lower dosage of dopaminergic medication but also possibly through a specific effect of STN DBS in the limbic circuit of motivation and reward [112].

## 6. Conclusions

In conclusion, there are very limited evidence on the efficacy of the treatment of GD symptoms in PD patients.

Management of GD in patients with PD under DA therapy is based on both patient and caregiver education (i.e., psychosocial interventions, such as counselling and cognitive behavioural therapy), modification of dopamine replacement therapy dosage to the lowest effective daily dose, or increase in L-dopa treatment and in some cases administration of topiramate or zonisamide, that up to date represent the only therapeutic option available in PD patients. Quetiapine and olanzapine represent a secondary line of therapy, although there are very few data supporting their role in PD.
